# Occupational voice demands and their impact on the call-centre industry

**DOI:** 10.1186/1471-2458-9-108

**Published:** 2009-04-20

**Authors:** DE Hazlett, OM Duffy, SA Moorhead

**Affiliations:** 1 School of Communication, University of Ulster, Shore Road, Newtownabbey, BT37 0QB, Northern Ireland; 2 School of Health Sciences, University of Ulster, Shore Road, Newtownabbey, BT37 0QB, Northern Ireland

## Abstract

**Background:**

Within the last decade there has been a growth in the call-centre industry in the UK, with a growing awareness of the voice as an important tool for successful communication. Occupational voice problems such as occupational dysphonia, in a business which relies on healthy, effective voice as the primary professional communication tool, may threaten working ability and occupational health and safety of workers. While previous studies of telephone call-agents have reported a range of voice symptoms and functional vocal health problems, there have been no studies investigating the use and impact of vocal performance in the communication industry within the UK. This study aims to address a significant gap in the evidence-base of occupational health and safety research. The objectives of the study are: 1. to investigate the work context and vocal communication demands for call-agents; 2. to evaluate call-agents' vocal health, awareness and performance; and 3. to identify key risks and training needs for employees and employers within call-centres.

**Methods and design:**

This is an occupational epidemiological study, which plans to recruit call-centres throughout the UK and Ireland. Data collection will consist of three components: 1. interviews with managers from each participating call-centre to assess their communication and training needs; 2. an online biopsychosocial questionnaire will be administered to investigate the work environment and vocal demands of call-agents; and 3. voice acoustic measurements of a random sample of participants using the Multi-dimensional Voice Program (MDVP). Qualitative content analysis from the interviews will identify underlying themes and issues. A multivariate analysis approach will be adopted using Structural Equation Modelling (SEM), to develop voice measurement models in determining the construct validity of potential factors contributing to occupational dysphonia. Quantitative data will be analysed using SPSS version 15. Ethical approval is granted for this study from the School of Communication, University of Ulster.

**Discussion:**

The results from this study will provide the missing element of voice-based evidence, by appraising the interactional dimensions of vocal health and communicative performance. This information will be used to inform training for call-agents and to contribute to health policies within the workplace, in order to enhance vocal health.

## Background

Research to date has failed to account for the central and most important tool for the communications industry, the human voice. Failure to investigate the use and impact of vocal performance has resulted in a significant gap in the evidence-base of occupational health and safety research. Voice is not simply the tool for communication transmission, it is directly related to the working ability and efficiency of the single most sought after attribute sourced by employers in the industry – effective verbal communication and interaction [1].

There is substantial epidemiological and physiological evidence supporting the view that groups such as teachers, singers, lawyers, and telemarketing workers are at risk of occupational health and safety voice problems [2,3], in particular, those working in call-centre environments [3]. Furthermore, majority of these studies include small participant numbers. The Department of Trade and Industry report [1] provided one of the most comprehensive reviews of the call-centre industry across the UK. Within the UK in 2003, there were 5,320 contact-centres and almost 500,000 agent positions, and this industry is forecast to continue to increase. A range of occupational safety and health (OSH) issues were identified as potential risks of current stressors in this work environment, raising the importance of regular appraisal and review of the impact of these issues on the health and economic well-being of the industry. Health and Safety Executive (HSE) [4] provided further focus, with an indepth investigation of stress and psychosocial stressors in the call-centre industry. Potential hazards were highlighted, related to vocal, optical, auditory and musculoskeletal health. Vocal health was evaluated by self-report of the frequency of symptoms, 'some, most or all of the time', with 39% reporting hoarseness; 32% pitch change and 42% discomfort in the throat. The report suggests that existing risks can be controlled through good work design and the provision of information and training. However, the contributory physiological demands have not been fully evaluated in relation to the physical work environment.

Vilkman [5] states that the prevention and treatment of occupational voice disorders required improved OSH arrangements. Vocal strain was classified as 'repeated movements (and collisions) of the vocal folds' as a potential repetition strain injury (RSI). The occupational medicine definition of RSI is 'overload of repetitive movements and static and dynamic muscle loading combined with biomechanical, ergonomic and psychological factors as well as poor treatment of early symptoms' [6]. As vocal abnormality refers to the person's ability to cope with the working tasks, reliable and valid tools for reporting on voice complaints are important [5].

Call-centre workers have prolonged voice use and heavy vocal loading, thus increasing the risk of occupational dysphonia, which is a voice disorder due to work-related overuse or abuse that threatens communicative, interactive and economic efficiency [2,5,7,8]. Vocal loading may be attributed to prolonged voice use, and the work environment, where background levels of voice may affect the quality, type and loudness of phonation in speech. Jones et al [9] compared the prevalence of voice problems among telemarketers compared to the general population and concluded that they were twice as likely to report one or more symptoms of vocal strain compared with controls. In addition, impaired work productivity due to voice problems occurred in 31% of the telemarketers surveyed. Lehto [10] identified risk factors in voice professions as: background noise, poor air quality (dryness, dust), poor posture and vocal loading.

Risks to vocal performance and efficiency are not simply physiological, but in telephone interaction, also demand co-ordination of optimum psychological, behavioural and environmental settings to maintain an efficient balance for the interaction. Clearly, subtle paralinguistic vocal changes may unconsciously affect the call-agent, but also the receiver negatively. Cameron [11] describes the demands of this work environment as emotional, cognitive and vocal. Furthermore, the focus of training and quality monitoring has been vocal styling and not skilling for the task. Lehto [12] suggests that when the call-agent's voice becomes strained, hoarse or effortful, this in turn adds physiological and cognitive stress which can result in less efficient interaction, particularly when following a script or protocol for calls.

Vast resources have already been spent without fully understanding or appreciating the interactional dimensions of telephone communication – physical, environmental, behavioural and psychosocial aspects of vocal communication. However, there have been no studies investigating the use and impact of vocal performance in the communication industry within the UK. This study aims to address this significant gap in the evidence-base of occupational health and safety research. This research will therefore provide the missing element of voice-based evidence, by appraising and ranking the interactional dimensions of vocal health and communicative performance.

The objectives of the study are:

1. To investigate the work context and vocal communication demands for call-agents;

2. To evaluate call-agents' vocal health, awareness and performance;

3. To identify key risks and training needs for employees and employers within call-centres.

## Methods and design

### Research design

This research will take the form of an occupational epidemiological study. This will be a mixed methods design, comprising a qualitative and large-scale quantitative approach, including acoustic measurement in the naturalistic work environment. There are three stages of this research study: 1. interviews with a senior manager (eg Call-centre or Human Resources Manager) within the call-centres; 2. online survey; and 3. acoustic measurements.

### Participants

The research participants will be employees from call-centres throughout the UK and Ireland. As this is a relatively new area of research, the main measurement tool, biopsychosocial questionnaire for the online survey will be developed to collect the relevant information to meet the aim and objectives of this study, thus power calculations were not calculated. The sample sizes of the participants in each stage of this study are as follows: 1. interviews with one manager from each participating call-centre (at least 10 call-centres); 2. online survey will be completed by a purposive sample of at least 500 call-agents from the participating call-centres; and 3. acoustic measurements will be conducted from at least 10% of participants from stage 2. It is anticipated that these sample sizes should be sufficient for the measurement tools to collect information on the vocal demands, communication, vocal health and training needs of call-agents in call-centres within the UK and Ireland. The findings from this study will be used to calculate a power calculation for the follow-up randomised controlled trial in call-centres.

The Human Resource department of each call-centre in the UK and Ireland will be contacted via e-mail inviting participation in this research study. Interested call-centres will contact the research team and receive full details of this study, and a manager from the participating organisations will provide written informed consent. The management team within the participating call-centres will inform their call-agents of this study and invite them to complete the online survey. The first question on the questionnaire for the online survey will ask the call-agents to indicate consent.

### Main measurement tool – biopsychosocial questionnaire

A biopsychosocial questionnaire will be developed for the online survey, and based on self-report tools, tested for reliability and validity. This questionnaire will be piloted among a sample of call-agents, different from those participating in the main survey. The questionnaire will include the Voice Symptom Scale (VoiSS) [13], Voice Handicap Index (VHI) [14], and The Vocology Screening Profile [15]. The VoiSS was developed from 800 participants and is psychometrically the most robust and extensively validated self report voice measure available [13]. This scale includes three domains of impairment, physiological and emotional aspects of voice symptoms and is assessed using a 5-item scale (0 = never and 4 = frequently). Example items are 'Do you feel you have to strain to produce voice?' and 'Does your voice make you feel incompetent?'. The VHI was devised by Jacobson and colleagues [14], and it uses a similar measurement 5-item scale. Example items are 'My voice difficulties restrict my personal and social life' and 'My voice problem upsets me'. In addition, the Vocology Screening Profile assesses vocal symptomatology [15]. This uses the same measurement scale and example items include 'Increased effort to talk' and 'Feeling thirsty', relating to physiological, acoustic and vocal function symptoms.

### Research procedures

For the first stage of this research, interviews will be conducted with a senior manager (eg Call-centre or Human Resources Manager) within the call-centres. These interviews will aim to assess the organisation's communication and training needs and will be via telephone or face-to-face, whichever is the most convenient for the manager. Current occupational safety and health roles will be identified and the analysis of this information will further inform the measurement tool. Secondly, a large scale epidemiology study will be conducted consisting of an online biopsychosocial questionnaire, which will be administered to investigate the work environment and vocal demands of call-agents. This will be published on a secured website and accessed at a convenient time, in agreement with the management team in the call-centres. Participants will receive information on the study including the time required for completion and assurance that all responses are anonymous. The third and final stage will involve acoustic measurement from a monitored telephone call-sample of a random sample of participants, who participated in the online survey. A sample of the natural conversation will be selected by a call-centre staff member and given to the research team in digital format for analysis.

### Data analysis

The data from the interviews will be transcribed and analysed thematically to determine context and process characteristics of each organisation. Qualitative content analysis will identify underlying themes and issues, which will be used to further refine and inform the questionnaire design.

Questionnaire analysis will account for the complex interaction of multi-factorial variables and the wide range of theoretical perspectives requiring evaluation with an appropriate range of methodological tools and approaches. Given the complexity of the study and the array of variables impacting on vocal strain, there is a need to employ sophisticated forms of analysis, with the capacity to explore potential directional effects across a wide range of variables. While multiple regression analysis is frequently used to analyse social science data, the main problem is handling measurement error, indirect or mediating effects. Since most multivariate procedures cannot deal with multiple variables, particularly multiple dependent or criterion variables, hypothesis testing is difficult.

Therefore, the study will adopt a multivariate analysis approach using Structural Equation Modelling (SEM), to develop voice measurement models in determining the construct validity of potential factors contributing to Occupational Dysphonia [16]. The research design and associated analysis will therefore attempt to overcome the traditional complications of multiple indicators, indirect effects and measurement error by using structural equation modelling with latent variables and LISREL (Linear Structural Relations) estimation. SEM is a statistical methodology that takes a confirmatory (ie hypotheses-testing) approach to the multivariate analysis of a structural theory bearing on some phenomenon, representing causal processes, which generate assumptions on multiple variables [15]. The hypotheses are: 1. Call-agents' physiological voice production is improved with good vocal-working environment; 2. Call-agents' acoustic voice production is improved with good vocal-working environment; 3. Call-agents' psychosocial health is improved with good vocal-working environment. The relationships and effects of these aetiological indicators on the 'risk' of occupational dysphonia will be explored in order to determine the relative significance of physiological, behavioural environmental and psychological indicators at varying levels of severity.

Acoustic analysis will be conducted on a random sample of the voice of call-agents from call-samples using the Multi Dimensional Voice Program (MDVP), which is a software programme that provides a robust multi-dimensional analysis of voice with graphic and numerical presentation of analysis [17]. MDVP is the gold standard measurement tool for quantitative analysis of voice. From the telephone call-samples, three sections each consisting of three seconds of the call-agent's continuous voice uninterrupted by the customer will be selected during 10 seconds at the first, middle and the end of the call. Three seconds of recording is within the recommended range for acoustic analysis by the manufacturer of the voice analysis software [17]. Therefore three sections (start, middle and end) of each call-sample will be used for acoustic analysis. The voices of the call-agents in these sample-calls will be analysed for the following 14 acoustic parameters: Mean Fundamental Frequency (MFo); Highest Fundamental Frequency (Fhi); Lowest Fundamental Frequency (Flo); Standard Deviation of Fo (STD); Amplitude Tremor Frequency (Fatr); Absolute Jitter (Jita); Jitter Percent (Jitt); Shimmer in dB (ShdB); Shimmer Percent (Shim); Peak-to-Peak Amplitude Variation (vAm); Noise to Harmonic Ratio (NHR); Degree of Voice Breaks (DVB); Degree of Sub-harmonics (DSH); and Degree of Voiceless (DUV). In addition, the acoustic results will be integrated into the voice measurement model using SEM.

All numerical data will be analysed using SPSS version 15. Descriptive statistics will include means, standard deviations and ranges for each variable from the online survey and acoustic analysis. Inferential statistics such as Pearson Correlations will be used to compare the objective and subjective measures. If the assumptions for the above parametric tests are violated then the appropriate non-parametric tests such as the Mann-Whitney will be used.

### Ethical issues

This study has obtained ethical approval from the School of Communication Risk and Ethics Filter Committee, University of Ulster. The questionnaires and the telephone call recordings will be anonymous. These will only be identified by code and not by individual call-agent's or customers' name. There are no known risks in this study.

### Data protection

All data will be stored securely, data including telephone recordings will be stored on password protected computers, and hard copies will be stored in a locked filing cabinet and will only be accessed by the research team. All information will be treated as confidential as per the Data Protection Act [18].

## Discussion

This study will investigate the work context and vocal communication demands for call-agents, and evaluates their vocal health, awareness and performance, and also identifies key risks and training needs for employees and employers within call-centres. Therefore, this study will provide the missing element of voice-based evidence, by appraising and ranking the interactional dimensions of vocal health and communicative performance.

The main body of evidence on occupational voice loss has been sourced from USA, Sweden, Poland, Finland and Australia. The definitions of occupational diseases differ from country to country. Dysphonia among teachers is considered as an occupational disease in countries such as France (only if the teacher is a state civil servant not a private teacher) and Russia but not in other countries such as Germany and the UK. In France, a simple dysphonia will not be over 25% of partial or permanent incapacity, and all related care and absenteeism will be 100% covered by the state. In Russia, if occupational dysphonia is confirmed by a medical team the treatment/re-education may be paid for by the Social Insurance Fund, and disability payment will be provided for the duration of the disability. In fact, in the UK, we are unable to assess the scale of the problem in working populations due to the occupational disease recording system, Occupational Physicians Reporting Activity (OPRA), with no cases of voice disorder recorded in any working population. The Industrial Injuries Advisory Council (IIAC) [19] considered the epidemiological evidence for risk of Occupational Voice Loss in those employed in occupations requiring high levels of voice use. The report concludes that there is an absence of good quality epidemiological data to indicate a doubling of risk of voice loss or vocal problems as a result of any particular type of work. Although a number of studies have been published these lack a consistent or objectively verifiable definition of the condition or of the exposure. It was concluded, therefore, that current evidence for occupational voice loss is insufficient to meet the requirements for prescription.

However a growing body of occupational voice research, not considered in the IIAC review [19], has provided further evidence on the early indicators and variables for investigation in measuring the range and levels of OSH hazards and risk. This study will provide a UK perspective, with the following benefits for employees and employers in call-centres and policy makers.

1. Benefits for Employees:

Increased awareness of the importance of preserving and enhancing the consistency and quality of their voice as the primary tool for communication.

2. Benefits for Employers:

Employers will not only fulfil their obligations under occupational health and safety legislation, but may also reduce days lost due to illness as a result of occupational dysphonia.

3. Benefits to health and OSH policies and practices:

Guidelines and recommendations for early detection and support for screening and training needs.

## Competing interests

The authors declare that they have no competing interests.

## Authors' contributions

DH and OD developed the original idea and were applicants on the successful funding application. All authors participated in development of the research protocol, and drafted all versions and approved the final manuscript.

## Pre-publication history

The pre-publication history for this paper can be accessed here:


